# Structural connectivity of the human olfactory network and its relation to aging and olfactory function

**DOI:** 10.1162/IMAG.a.1181

**Published:** 2026-03-27

**Authors:** Xin Li, Jonas Olofsson, Jonas Persson

**Affiliations:** Aging Research Center, Karolinska Institute and Stockholm University, Stockholm, Sweden; Centre for Sleep & Cognition & Centre for Translational Magnetic Resonance Research, Yong Loo Lin School of Medicine, National University of Singapore, Singapore, Singapore; Department of Psychology, Stockholm University, Stockholm, Sweden; Center for Lifespan Developmental Research (LEADER), School of Behavioral, Social and Legal Sciences, Örebro University, Örebro, Sweden

**Keywords:** olfaction, episodic memory, aging, structural connectivity, primary olfactory cortex, diffusion weighted imaging

## Abstract

Impaired olfactory function in older adults is associated with memory decline and is a biomarker of Alzheimer’s disease (AD). However, the structural brain foundation underlying olfactory impairment and its link to memory function remains largely unknown. We address this gap by reconstructing the structural olfactory network, that is, white-matter connections between the primary olfactory cortex (POC) and the whole brain. Through applying multivariate analyses in a population-based sample (n = 137), we investigate the relationships among age, the olfactory network, and olfactory and cognitive function. Our findings reveal that the POC subregions have distinct structural connectivity profiles with the entire brain. Older age was associated with weaker connectivity strength between the POC and nearby regions, suggesting a reorganization of the olfactory network in older adults. Structural connectivity of the olfactory network was associated with behavioral performance in odor identification, episodic memory, and odor threshold, but not processing speed or working memory. Notably, connections including the olfactory tubercle (TUB)-caudate, TUB-amygdala, and olfactory nucleus (AON)-hippocampus were important for both olfaction and episodic memory function, suggesting a common neural basis across cognitive domains. Our study expands on previous research of single brain regions or individual white-matter tracts, uncovering the structural underpinnings of olfactory function at the network level. The results shed light on the common foundation of olfaction and memory dysfunction, an early marker of AD.

## 1 Introduction

Olfaction, the sense of smell, is one of the phylogenetically oldest senses, allowing animals to find food and avoid predators. In humans, olfaction is often regarded as less important than vision or hearing (Enoch et al., 2019). However, decline of olfactory function may signal broader health issues. In older adults, olfactory loss further predicts faster cognitive decline in older adults (Devanand et al., 2015; Dintica et al., 2019; Olofsson et al., 2009; Schubert et al., 2008), and an increased risk of conversion to dementia (Motter et al., 2023; Murphy, 2019; Stanciu et al., 2014). Olfactory processing starts when odorant molecules bind to olfactory sensory neurons in the nose with their axons projecting to the olfactory bulb. The primary olfactory cortex (POC) receives direct input from the olfactory bulb (Haberly, 2001; Shipley & Ennis, 1996). The detection, identification, and categorization of olfactory stimuli, and their further involvement in cognition and emotion occur in the interaction between the POC and associated regions that constitutes the olfactory network (Gottfried, 2010). We asked how the structural olfactory network is influenced by chronological age, and if age-related differences in the network are linked to olfactory and cognitive performance decline in aging.

The most well-established human POC consists of the anterior olfactory nucleus (AON), the olfactory tubercle (TUB), and the frontal and temporal piriform cortices (PirF, PirT). Unlike in rodents, there is currently no evidence that entorhinal cortex receives direct projections from the olfactory bulb in humans and thus the entorhinal cortex is not considered part of the primary olfactory cortex (Echevarria-Cooper et al., 2022). Previous functional MRI studies have shown that the POC subregions have different functional connectivity with the rest of the brain (Zhou et al., 2019, 2021), suggesting that they contribute to distinct functional olfactory networks. Functional connectivity is measured by the temporal correlation between pairs of brain regions, and therefore does not necessarily reflect underlying axonal connections. Previous diffusion tensor imaging (DTI) studies have demonstrated that the POC was structurally connected to multiple brain regions, including the orbitofrontal cortex, rectus gyrus, parahippocampus, striatum, and temporal cortex (Fjaeldstad et al., 2017, 2021). However, these studies did not distinguish between POC subregions. Moreover, they used a tensor-based white-matter model, which is a voxel-level measure of white-matter integrity that does not estimate multiple crossing white-matter fibers of different directions within the voxel. Thus, it provides a limited method for constructing complex structural connectomes. For example, the POC-hippocampus connection that has repeatedly been found in human functional studies (Zhou et al., 2019, 2021), and non-human animal work (Aqrabawi et al., 2016; Levinson et al., 2020) could not be identified using DTI. We addressed these research gaps by using the constrained spherical deconvolution (CSD, Tournier et al., 2004, 2007) white-matter model, which can separate white-matter fibers of different orientations within the same voxel. To provide a detailed structural brain network of olfaction, we generated the POC subregions based on a previous template (Zhou et al., 2019) and identified structural connections between the POC subregions and the rest of the brain.

Human olfactory function is commonly assessed using tests of odor identification (identifying names of odors in a multiple-choice format) and odor threshold (detecting low concentrations of an odor stimulus from a no-odor background). Older individuals are typically impaired in their olfactory abilities, and reduced olfactory function predicts a faster rate of cognitive decline (Dintica et al., 2019; Schubert et al., 2008). It has also been suggested that olfactory deficit may be an early marker for Alzheimer’s disease (Devanand et al., 2015; Murphy, 2019). While numerous studies have focused on the consequences of olfactory impairment on health and aging, relatively few studies have examined the underlying brain mechanisms of these impairments. Some studies have found that gray matter volumes of the hippocampus (Dintica et al., 2019; Menelaou et al., 2022), temporal lobe (Dintica et al., 2019; Jones-Gotman & Zatorre, 1988), entorhinal cortex (Dintica et al., 2019), and amygdala (Segura et al., 2013) were associated with odor identification performance. Others, however, have not found such associations (Fjaeldstad et al., 2021; Smitka et al., 2012). This inconsistency may be because olfactory function primarily relies on complex interaction of multiple brain regions within the olfactory networks, rather than the involvement of one or a few separate brain regions.

Older adults typically exhibit heterogeneous changes in structural connectivity across the brain, including a greater decline in white-matter fiber densities in hub connections compared to peripheral connections in older adults (Li et al., 2023). These variations may lead to topographical alterations at the whole-brain level, such as a more integrated and less segregated brain network organization in older adults (Chan et al., 2014; Pedersen et al., 2021). While we recently demonstrated that change in olfactory detection was related to change in corpus callosum white-matter using DTI (Li et al., 2025), it is largely unknown how the structural connectivity of the olfactory network changes with aging. This knowledge is critical for understanding the mechanisms of age-related olfaction impairment in older adults.

By applying the CSD white-matter fiber model and multivariate data analysis, the current study addresses important questions regarding the anatomical organization of olfactory function. By using a large sample with a wide age range (25–85 years), we (1) explored the structural organization of the olfactory networks, (2) examined the age effects on the structural olfactory networks, and (3) assessed if these age effects can explain behavioral impairments in olfactory and cognitive function in older adults.

## 2 Methods

### 2.1 Study sample

Participants in this study belong to the Swedish Betula study, a longitudinal cohort study on memory, health, and aging (Nilsson et al., 1997; Nyberg et al., 2020). The Betula study was reviewed and approved by the local Regional Ethical Vetting Board at Umeå University. The research was conducted in accordance with the ethical principles regarding human experimentation outlined in the Declaration of Helsinki. All participants were provided written informed consent in accordance with the guidelines of the Swedish Research Council. Using a narrow age cohort (NAC) design, the researchers behind the Betula project recruited participants at specific ages in the range of 35–80 years, with a 5-year interval (i.e. 35, 40, 45, etc). The NAC design decreases confounding effects such as education and nutritional level (Rönnlund et al., 2005; Sternäng et al., 2008). In the present work, we excluded participants with dementia, stroke, epilepsy, Parkinson’s disease, multiple sclerosis, hydrocephalus, head surgery, deviant brain morphology, and vascular brain lesions (white-matter hyperintensities and brain infarcts). Vascular lesions were identified from visual inspection of the structural T1- and T2-FLAIR images by a trained neuroradiologist. White-matter lesions were defined as larger focal hyperintensities on T2-weighted images. Infarcts were classified as abnormalities located in the white matter and had to be hypointense on T1-weighted and hyperintense on FLAIR images to distinguish them from smaller white-matter lesions. Imaging data with severe geometric distortion across the entire brain were excluded from the analysis. Dementia was assessed at baseline and reassessed for each wave using a three-step procedure, according to the Diagnostic and Statistical Manual of Mental Disorders, 4th edition (DSM–IV; American Psychiatric Association, 1994). First, an overall evaluation was performed by an examining physician. The diagnosis was then compared with a second independent diagnosis based on scores from several cognitive tests. In cases of disagreement, a supervising physician made a third and final diagnosis. In the baseline sample, the scanning parameter echo time (TE) varied in some participants. Since TE influences the estimated white-matter (WM) fiber densities, 70 participants with different TE were excluded. Our final sample included 137 healthy older adults without dementia (mean age = 62 years, age range: 25–85 years). The demographic information and task performance of the study samples are shown in Table 1. It should be noted that sex is unevenly distributed in the current sample. However, as the effect of sex is not the focus of this study, and including sex as an additional covariate did not change the pattern of results, it was not included in the analysis.

**Table 1.** Demographic, cognitive, and olfactory variables in the study sample, which was divided into three age cohorts.

Mean (standard deviation)	25–40 year	45–60 years	> = 65 years
Females/males	7/11	11/33	19/56
Education in years	15.61 (2.45)	13.9 (3.05)	11.86 (4.57)
Odor identification	8.44 (1.89)	7.02 (2.14)	6.22 (1.74)
Odor threshold	7.78 (2.96)	5.43 (2.1)	5.78 (2.66)
Episodic memory	46.39 (8.75)	43.72 (7.86)	38.15 (8.93)
Processing speed	36.89 (8.78)	32.47 (5.15)	26.2 (7.16)
Working memory	35.56 (3.38)	33.95 (3.55)	30.98 (4.81)

### 2.2 Olfactory assessments

Two olfactory tests, odor identification and odor threshold, were included in the study. The odor identification test is a revised version of the Scandinavian Odor Identification test (SOIT, Nordin et al., 1998), which is a 16-item, 4-alternative forced-choice test. In the current version, 3 odorants that had a trigeminal impact were removed from the original test. The remaining 13 odorous stimuli included: vanilla, lemon, apple, violet, orange, anise, tar, clove, bitter almond, cinnamon, pine-needle, lilac, and juniper berry (Larsson et al., 2004). The response options were also modified to include more similar alternatives, which increased the task difficulty to prevent a ceiling effect (Nordin et al., 1998). The included odors are relatively strong in intensity and are supposed to represent a wide range of exposures in real life. Participants had a 30 s interval between stimuli to avoid perceptual adaptation. The scores on the modified SOIT used in the Betula study predict future risk of cognitive decline (Olofsson et al., 2009), dementia (Stanciu et al., 2014), and mortality (Ekström et al., 2017). Odor detection thresholds were measured using ‘Sniffin’ Sticks,’ which contain n-butanol at varying concentrations across 16 dilution steps, starting with the intermediate concentration step 8. Participants, with eyes closed, were presented with two pens—one containing an odorant and the other a blank—in random order. They identified which pen had the odor. If correct, the same concentration was retested until four consecutive correct identifications were made, then trials proceeded at the lowest concentration, step 16. If incorrect responses were made at step 8, new trials proceeded at stronger odor concentrations, starting with step 7 and continuing with increasing concentrations until four consecutive correct responses were obtained. The weakest concentration detected was recorded as their score, with higher scores indicating better odor detection.

### 2.3 Other cognitive measurements

We examined whether olfactory network structural connectivity is related to episodic memory, working memory, and processing speed. Whole-brain white matter integrity is associated with processing speed and working memory (Li et al., 2022, 2023). Moreover, olfactory function has been associated with episodic memory (Dintica et al., 2021), likely due to shared neural substrates (Arshamian et al., 2013). Thus, we investigated whether the olfactory network specifically relates to episodic memory or reflects general cognitive function. Briefly, episodic memory was assessed as the composite scores from five tests: immediate free and delayed cued recall of sentences (under both enacted and non-enacted conditions), and immediate free recall of unrelated nouns. Working memory was measured using the 2-back task. During this task, words were presented orally to participants, who were required to determine whether the current word matched the one presented two positions earlier in the sequence. For processing speed, we used the composite score derived from the letter-digit substitution, pattern comparison, and letter comparison tasks (see Nilsson et al., 1997 for more details).

### 2.4 Image acquisition and preprocessing

Diffusion-weighted imaging (DWI) data were collected on a 3T Discovery MR750 (General Electric) scanner with a 32-channel head coil. Data were acquired using a single-shot, spin-echo-planar, T2-weighted sequence, with a spatial resolution of 0.98 × 0.98 × 2 mm. The sequence parameters were: TR = 8.0 s, TE = 84.4 ms, 64 slices with no gap in between, 256 × 256 matrix (FOV = 250 mm), 90º flip angle, b = 1000 s/mm^2^, and six b = 0 images. Three DWI sessions of 32 independent directions were included for each participant. T1-weighted images were acquired with a 3D fast spoiled gradient echo sequence (TR: 8.2 ms, TE: 3.2 ms, field of view: 25 × 25 cm, flip angle: 12°, 180 slices with a 1 mm thickness). T2-weighted Fluid-Attenuated Inversion Recovery (FLAIR) images were acquired with a 2D T2 FLAIR sequence (48 slices with 3 mm thickness; TR: 8000 ms, TE: 120 ms, field of view: 24 × 24 cm). All included participants were examined on the same scanner with no software or hardware updates during the data-collection period.

The raw DWI data were first pre-processed using MRtrix 3.0, including denoising, unringing and motion correction, topup, eddy current correction, and bias field correction. Synb0 (Schilling et al., 2019, 2020) was used to correct for spatial distortions caused by susceptibility-induced off-resonance fields. This deep-learning approach produces corrected images that closely align with undistorted anatomical images (See Supplementary Fig. S1 for an example) and has been shown to perform comparably to ‘Top-up’ correction (Schilling et al., 2019). We used the CSD (Tournier et al., 2004, 2007) to obtain the Fiber Orientation Distributions (FODs) in each voxel. This was done by first estimating the response functions for single-fiber WM as well as gray matter (GM) and cerebro-spinal fluid (CSF) using an unsupervised method (Dhollander et al., 2019). Single-Shell 3-Tissue CSD (SS3T-CSD) was performed to obtain WM-like FODs as well as GM-like and CSF-like compartments in all voxels (Dhollander & Connelly, 2016), using MRtrix3Tissue (https://3Tissue.github.io), a fork of MRtrix3 (Tournier et al., 2019). This approach gives similar results as multi-shell data (Dhollander & Connelly, 2016). WM FOD images were then corrected for intensity inhomogeneities and used in subsequent analyses.

To align diffusion and T1-weighted images for each individual, boundary-based registration (BBR) was used to estimate the rigid transformation matrix from B0 to T1-weighted images while fitting the white-matter boundary in FSL. This approach is more robust to pathologies and artefacts in the EPI images (Greve & Fischl, 2009). The output matrix was then applied to transform T1-images to diffusion images.

Whole-brain Anatomically Constrained Tractography (ACT, Smith et al., 2012) was performed on the WM FOD images. ACT is an additional module to the streamlines tractography, which uses aligned T1-images as anatomical priors to constrain the streamlines beginning, traversing, and ending in anatomically plausible regions. This was achieved by processing and segmenting the T1 images into 5 different tissue types, cortical GM, sub-cortical GM, WM, CSF, and pathological tissue. The segmented images with 5 different tissues were then fed into the whole-brain tractography as a mask. ACT improves the biological accuracy of tractography and greatly decreases false-positive streamlines. Ten million tractography streamlines were reconstructed, with the GM–WM interface used as seed regions. The generated tractograms were filtered using spherical-deconvolution informed filtering of tracks (Smith et al., 2015). All diffusion preprocessing and tractography steps were performed in MRtrix3 using default or recommended parameters, unless otherwise specified. Quantitative quality control metrics were derived using FSL’s eddy_qc framework. Across all 137 participants, mean absolute head motion was 0.57 ± 0.32 mm (range 0.17–2.41 mm), mean relative motion was 0.25 ± 0.06 mm (0.19–0.80 mm), and the percentage of slices classified as outliers was 0.13 ± 0.24% (0.00–1.76%). In addition, each intermediate output (raw diffusion images, response functions, fiber orientation distributions, registration, and tractograms) was visually inspected for quality, and participants with clear artefacts were excluded from further analysis.

We used the AAL atlas to segment the whole brain into 45 regions. The AAL atlas defines larger regions of interest (ROIs), reducing the influence of noise and processing artifacts such as misalignment and geometric distortion. Additionally, it is a commonly used volumetric atlas with anatomical information, allowing us to interpret the results in a localized manner and compare them with findings from non-human studies on the POC. The AAL atlas, which is originally in MNI space, was registered to native space of the T1-weighted images for each individual using Advanced Normalization Tools (Avants et al., 2014).

The cerebellum and the brainstem were not included in some images because of the restricted field of view. We parcellated the POC into 4 subregions, AON, TUB, PirF, and PirT (Supplementary Fig. S2) using the atlas from Zhou et al. (2019). Structural connectomes between the 4 subregions and the entire brain were constructed using tractograms and the parcellation atlas. Fiber bundle capacity (FBC, Smith et al., 2022), which is a weighted sum of streamlines, was used to estimate the structural connectivity strength/edges of the network. The SIFT2 algorithm assigns a weight for each streamline iteratively, so that the weighted sum matches mean fiber densities between pairs of ROIs in white-matter FOD images. Compared to the commonly used connectivity measures, such as the raw number of streamlines, FBC is reflective of the underlying biological connectivity, that is, fiber density (Smith et al., 2022) and is therefore more robust and less influenced by brain volume or streamline length. We did not have any a-priori hypothesis regarding lateralization within the POC, thus the values from the two hemispheres were averaged to increase statistical power and further reduce noise introduced by processing. This step resulted in a 4 × 49 matrix for each participant. Since the distribution of FBC is heavily right-skewed, we used the log-transformed FBC to quantify the connection strength of the network in the analysis. We also defined a reference network with only strong connections to the POC based on the younger sample of 25–45 years. We implemented a commonly used two-step criteria procedure to select connections to be included in this network (Arnatkeviciute et al., 2021): (i) present in at least 80% of participants; and (ii) among the 30% strongest edges of the mean connectome. This strongly-connected network was only used as a reference in Figures 2–5 for illustrative purposes.

### 2.5 Statistical analysis

We investigated the relationship between the olfactory network and age/behavior using kernel ridge regression (KRR) from an open-source pipeline (Chen et al., 2022). KRR is a multivariate analysis technique that incorporates all the edges of the brain network into the regression model through L2 regularization, which has better model performance than other regularizations, such as lasso and elastic net (Gao et al., 2019). KRR computes the similarities between x variables using kernel functions to predict y variables, assuming that individuals with similar brain features exhibit similar age or behavioral performance. It performed comparably to deep neural networks in predicting demographic and behavioral outcomes from the brain connectome (He et al., 2020). We used KRR to predict age and olfactory/cognitive measures based on brain structural connectivity features. The input feature space consisted of FBC values connecting the 4 POC subregions to the rest of the brain (48 regions), yielding a total of 192 features per subject. Following the methodology of Chen et al. (2022), prediction performance was evaluated using a 3-fold nested cross-validation (CV) framework. In the outer loop, the entire sample was randomly partitioned into a training set (N = 92) and a test set (N = 45). The training set was then used for hyperparameter tuning via an inner loop (3-fold CV). Specifically, within the inner loop, the training set was further split into training and validation folds to select the optimal L2 regularization parameter based on validation performance. The hyperparameter yielding the highest accuracy in the inner loop was subsequently applied in the final model. Pearson’s correlation and Mean Absolute Error (MAE) were used to estimate the predictive performance on the held-out test set. To ensure the robustness of the results and minimize the bias of random data partitioning, this entire nested CV procedure was repeated 100 times. Since there are significant negative age effects on both FBC and olfaction/cognition, age was included as the covariate in predictions of olfactory/cognitive function from brain connectome.

To interpret how each connection contributes to the models, we measured the importance of each edge in the olfactory network to the prediction models using Haufe’s inversion approach (Chen et al., 2022; Haufe et al., 2014). A positive (or negative) predictive-feature value indicates that stronger (or weaker) structural connectivity was associated with larger behavioral outcomes or older age.

Permutation tests were performed to examine if the model performance and the predictive-feature values deviated from the chance level. We obtained the two-tailed P-values by shuffling variables of behavioral outcomes and age 1000 times. P-values were defined as the proportion of permutations in which the metric was high/lower than the value obtained with the true, unshuffled data. For each shuffling, we used the optimal hyperparameters that were already generated based on the true data and thus only the 3-folded outer loop of CV will be repeated.

We applied False Discovery Rate (FDR) correction to account for multiple comparisons. The correction was performed separately for three sets of analyses: (1) the prediction accuracy of age and behavioral measures (6 tests), (2) the significance of individual connectome features (192 tests), and (3) the spatial correlations between feature importance maps (12 tests).

To investigate if the spatial patterns of the connections that predict behavioral and age are similar, we correlated predictive-feature values between behavior and age. 1000 randomly shuffled brain maps (surrogate maps) with preserved similar spatial autocorrelation (SA) were generated using BrainSMASH (Burt et al., 2020). Pearson correlations were performed between brain map of the predictive-feature values and 1000 randomly shuffled brain maps.

## 3 Results

**Fig. 1.**
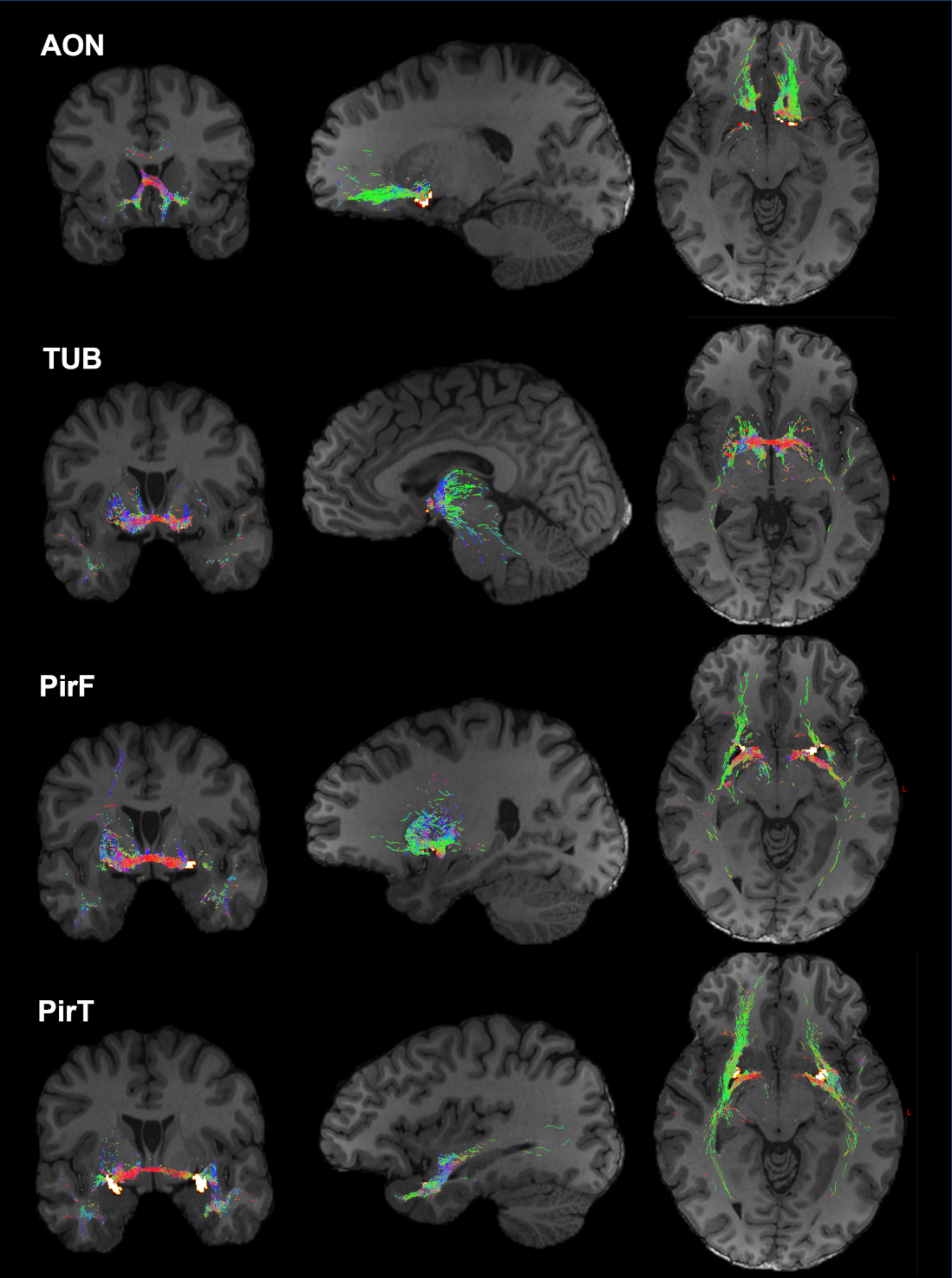
White-matter connection (tractogram) from 4 subregions of the primary olfaction cortex (POC), anterior olfactory nucleus (AON), olfactory tubercle (TUB), frontal piriform cortices (PirF), and temporal piriform cortices (PirT), to the rest of the brain. Color represents fiber orientation. The tractogram is based on the DWI from 1 25-year-old participant for illustrative purposes.

We reconstructed the structural connectivity between the 4 POC subregions and the entire brain. All 4 subregions had strong connections with the hippocampus, striatum, amygdala, and thalamus. Moreover, the subregions had unique connectivity patterns to the rest of the brain (Fig. 1). PirT had much stronger connections to the temporal lobe, especially its anterior parts, compared to other subregions. AON showed stronger connections to the inferior and superior parts of the frontal orbital cortex and rectus gyrus. TUB was especially connected to the pallidum. PirF had generally weak connections to the rest of the brain, being mainly connected to the pallidum, the insula, and the temporal pole. Moreover, the POC had generally stronger connections with the inferior areas of the brain that are anatomically closer to the POC compared to the superior areas of the brain that are more distant from the POC. We assessed the relation between Euclidean distance and connectivity strength. Results generally demonstrated that shorter-range connections were strongly connected to the POC and longer-range connections were relatively weaker (Supplementary Fig. S3). This association was not driven by the systematic streamline-length bias induced during tractography. We used FBC together with the SIFT2 algorithm to quantify the connectivity strength. FBC is a normalized measure that only reflects the estimation of white-matter density between endpoints; thus it is independent from the length of white-matter fiber bundles compared to other connectivity measures (Smith et al., 2022).

**Fig. 2.**
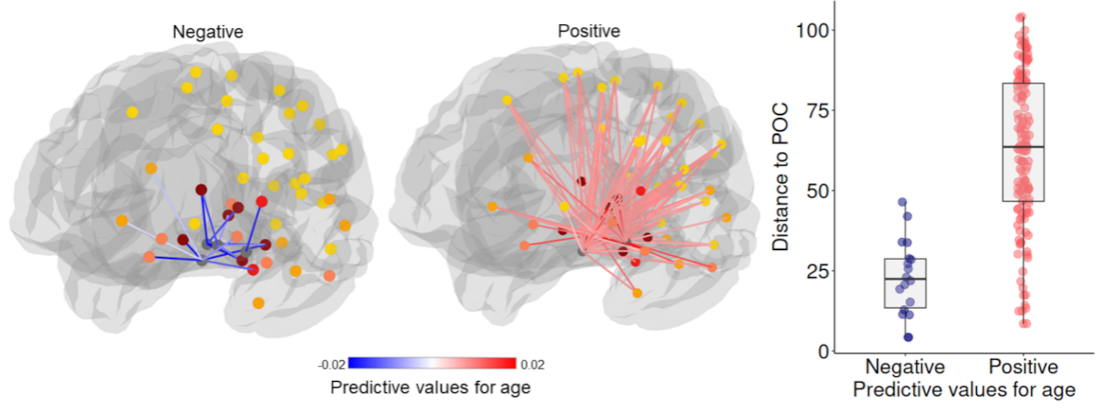
Structural connectivity supporting individual-level age prediction. Network (left) displays connections from four POC subregions (grey nodes) to the rest of the brain. Edge colors represent predictive-feature values: positive values in red indicate that higher FBC is associated with older age, negative values in blue indicate the negative association. Node colors represent the number of connections (0–4; yellow to dark red) to POC subregions within this strongly connected network. While the main analysis included the fully connected olfactory network, this visualization displays the strongly connected subnetwork structure (See methods for more details regarding how the network is defined). The scatter plot (right) shows the Euclidian distance to the POC between positive and negative predictive edges.

We then investigated age effects on the olfactory network. FBC of the olfactory network predicted age with high accuracy (r = 0.583, p = 0.001, FDR q < 0.05). The model achieved an MAE of 10.13 years (age range: 25–95 years), significantly better than chance (p = 0.001, FDR q < 0.01). We also observed significant positive weights (FDR q < 0.05) of the predictive features for age (Fig. 2; See Supplementary Fig. S4 for the labels of the significant connections).

We examined if the FBC of the structural olfactory network could predict both olfactory and cognitive functions. Our results show that, after controlling for age, odor identification (score range: 0–12) was significantly predicted (r = 0.155, p = 0.019, FDR q < 0.05; MAE = 2.057, p = 0.03, FDR q = 0.054). Episodic memory (score range: 0–72) also yielded significant prediction accuracy (r = 0.193, p = 0.004, FDR q < 0.05; MAE = 8.672, p = 0.018, FDR q = 0.054). Odor threshold (score range: 0–16) showed a non-significant correlation (r = 0.097, p = 0.101 FDR q > 0.05) and MAE of 2.58 with marginal significance (p = 0.036, FDR q = 0.054). The prediction models for processing speed (r = 0.122, p = 0.052, FDR q > 0.05; MAE = 7.757, score range: 0–50, p = 0.537, FDR q > 0.05) and working memory (r = -0.04, p = 0.672, FDR q > 0.05; MAE = 4.222, score range: 0–40, p = 0.23 FDR q > 0.05) were not significant.

**Fig. 3.**
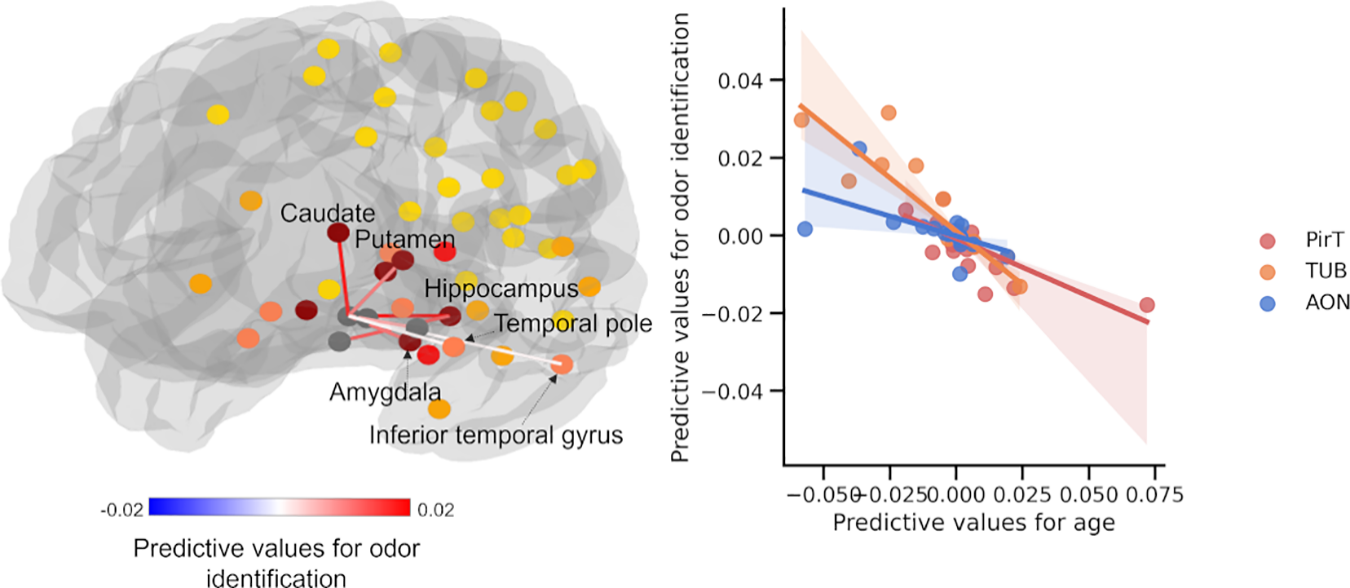
Structural connectivity supporting individual-level prediction of odor identification. Network (Left) where node colors represent the number of connections (0–4; yellow to dark red) to POC subregions within the strongly connected network. Scatter plot (right) showing the linear relationship between predictive-feature values for age and for odor identification. Each point represents a structural connection between two ROIs. Positive feature values indicate that higher FBC is associated with greater age or better odor identification performance. POC subregions showing non-significant predictive correlations (P^perm^ > 0.05) of predictive values were omitted for clarity.

**Fig. 4.**
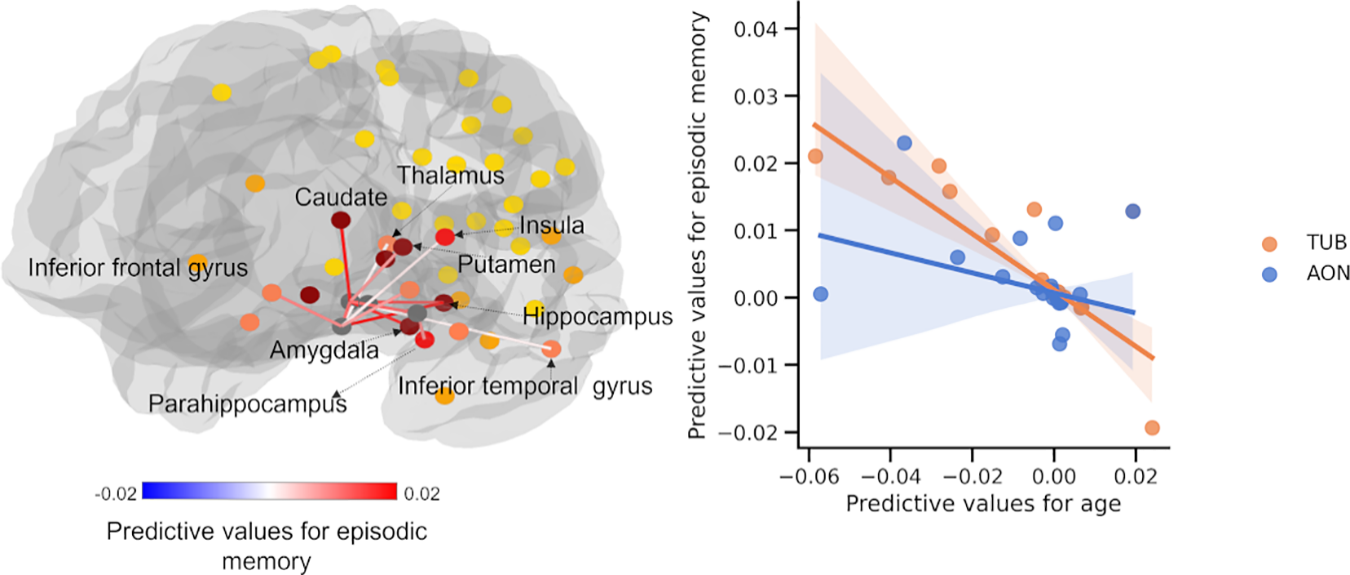
Structural connectivity supporting individual-level prediction of episodic memory. Network (Left) where node colors represent the number of connections (0–4; yellow to dark red) to POC subregions within the strongly connected network. Scatter plot (right) showing the linear relationship between predictive-feature values for age and for episodic memory. Each point represents a structural connection between two ROIs. Positive feature values indicate that higher FBC is associated with greater age or better memory performance. POC subregions showing non-significant predictive correlations (P^perm^ > 0.05) of predictive values were omitted for clarity.

**Fig. 5.**
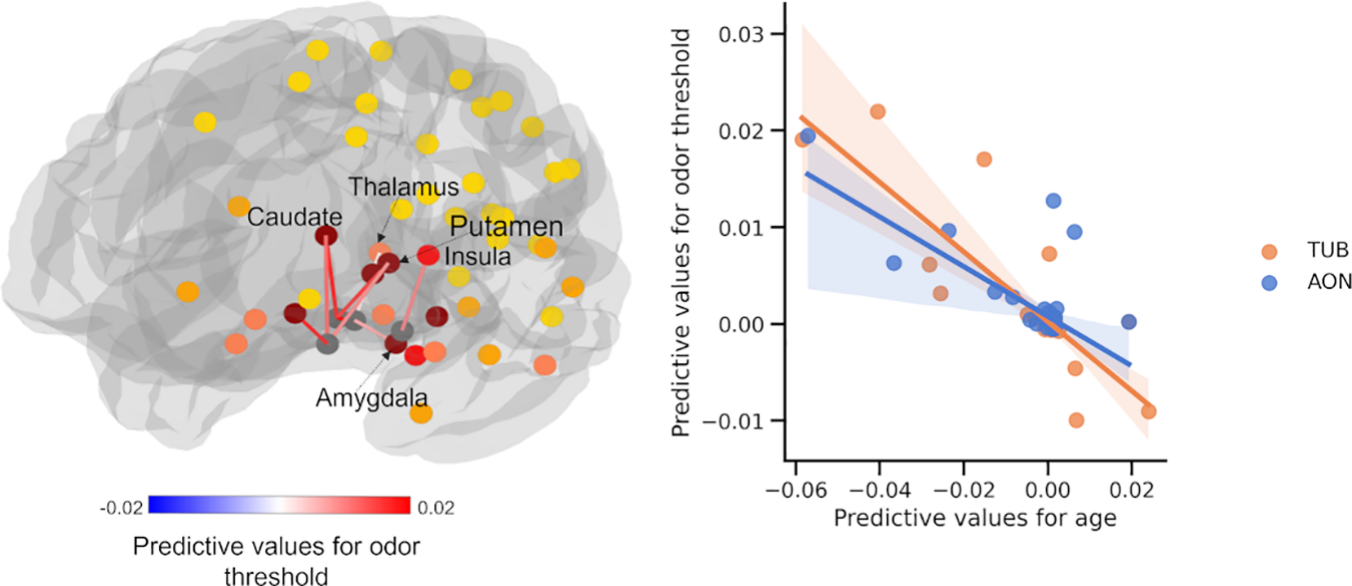
Structural connectivity supporting individual-level prediction of odor threshold. Network (Left) where node colors represent the number of connections (0–4; yellow to dark red) to POC subregions within the strongly connected network. Scatter plot (right) showing the linear relationship between predictive-feature values for age and for odor threshold. Each point represents a structural connection between two ROIs. Positive feature values indicate that higher FBC is associated with greater age or lower odor threshold (more sensitive olfaction). POC subregions showing non-significant predictive correlations (P^perm^ > 0.05) of predictive values were omitted for clarity.

For odor identification, we found that the larger FBC between TUB to the hippocampus, caudate, putamen, amygdala, and temporal lobe, connections between AON and the hippocampus, connections between PifT and caudate, and interconnections within the POC predicted higher performance (FDR q < 0.05, Fig. 3). For episodic memory, the connections predicting task performance included those between the PirT and parahippocampus; the TUB and the amygdala, hippocampus, parahippocampus, caudate, and putamen; the AON and the inferior/superior orbital frontal cortex and hippocampus; and within the POC. (FDR q < 0.05, Fig. 4). For odor threshold, greater sensitivity to odors (lower threshold) was associated with stronger connections between the PirT and insula; the PirF and amygdala and thalamus; the TUB and caudate and putamen; the AON and caudate and putamen; as well as connections within the POC (FDR q < 0.05, Fig. 5).

The feature-importance map of episodic memory partly overlapped with that of odor identification, in the connections of TUB and the amygdala, caudate, and putamen, and connection between AON and the hippocampus. The results demonstrate that these connections may be critical for both episodic memory and odor identification. There was also an overlap in the feature-importance map between episodic memory and odor threshold, i.e., the objective perception of smell. This overlap included connections between the TUB and the caudate, as well as the TUB and the putamen.

We further found that the spatial patterns of feature importance were correlated among age, olfactory function, and episodic memory (scatter plots depicted in Figs. 3, 4, and 5). Due to the distinct connectivity profiles of the POC subregion, the analysis was conducted for the four subregions separately. The results showed that, after controlling for spatial autoregression, the correlations between age and odor identification were significant for AON (r = -0.786, p < 0.001, FDR q < 0.05), PirT (r = -0.516, p < 0.001, FDR q < 0.05), and TUB (r = -0.673, p < 0.001, FDR q < 0.05), but not for PirF (r = -0.216, p = 0.132, FDR q > 0.05). The correlations between age and odor threshold were significant for AON (r = -0.484, p = 0.004, FDR q < 0.05) and TUB (r = -0.565, p < 0.001, FDR q < 0.05), but not for PirT (r = -0.132, p = 0.434, FDR q > 0.05) and PirF (r = -0.238, p = 0.211, FDR q > 0.05). The correlations between age and episodic memory were significant for AON (r = -0.63, p < 0.001, FDR q < 0.05) and TUB (r = -0.633, p < 0.001, FDR q < 0.05), but not for PirF (r = -0.043, p = 0.797, FDR q > 0.05) and PirT (r = -0.359, p = 0.134, FDR q > 0.05).

Finally, structural connectivity still can predict odor identification performance while controlling for both age and episodic memory (r = 0.105, p = 0.07; MAE = 2.01, p = 0.042). Also, when additionally controlling for episodic memory, the correlations between the feature importance of age and odor identification were still significant for AON (r = -0.684, p < 0.001), PirT (r = -0.539, p = 0.002), and TUB (r = -0.68, p < 0.001), but not for PirF (r = -0.208, p = 0.226) (Supplementary Fig. S5). These findings imply that odor identification reflects a composite of perceptual and cognitive processes, which cannot be fully disentangled merely by controlling for single cognitive covariates (e.g., episodic memory).

## 4 Discussion

In this study, we reconstructed the structural connectome of the olfactory network using a CSD white-matter model, where the connectivity strength reflects the white-matter fiber density between pairs of brain regions. We found that the POC strongly connects to the limbic system, temporal lobe, striatum, and orbital frontal cortex. The POC subregions have overlapping, but also partly distinct structural connectivity profiles with the rest of the brain. These findings are consistent with, and extend previous results of, functional connectivity results in humans (Zhou et al., 2019). Based on a study sample of adult participants with a wide age range, we were able to demonstrate negative age effects on the structural connectivity between POC and the hippocampus, striatum, amygdala, and frontal orbital and temporal lobe that have smaller Euclidian distance with POC. Results also showed positive age effects in terms of stronger connections to the posterior part of frontal, parietal, and temporal brain regions that are more distant from the POC. These results may reflect age-specific patterns in olfactory network connectivity. By directly linking the structural connectome to behavioral performance, we found that the structural olfactory network can predict performance on both odor identification and episodic memory with accuracy significantly better than chance level. Maps of the feature importance between the age effects and the associations with both olfactory function and episodic memory are highly correlated, providing evidence that age-related alteration of the olfactory network may partly explain impaired olfaction and episodic memory in older adults. Our present work thus complements research that has indicated aging effects in the epithelium, or hippocampal volume loss, as key drivers of odor identification impairment (Doty et al., 2011; Menelaou et al., 2022). Moreover, we demonstrate that the TUB-caudate, TUB-amygdala, and AON-hippocampus connections are important for both odor identification and episodic memory, which indicates a common structural underpinning of these two cognitive domains. Our findings have implications for the structural basis of olfaction in aging and suggest that the olfactory network may serve as a key neural correlate of age-related decline in olfactory function and memory.

### 4.1 The POC subregions have distinct structural connectivity profiles with the rest of the brain

Within the POC subregions, the piriform cortex is the largest area and is most frequently studied. The piriform cortex receives direct input from the olfactory bulb (Stettler & Axel, 2009) and have widespread projection to multiple cortical areas (Calu et al., 2007; Johnson et al., 2000; Mesulam, 1998). Human fMRI studies found that PirT and PirF have distinct functional connectivity patterns (Zhou et al., 2021). Thus, they may have complementary functional roles such that PirF encodes odorant’s chemical structure, whereas PirT encodes odor-object identity (Gottfried et al., 2006). A study using neurophysiological recording and anatomical tracing demonstrated that in rats, anterior piriform cortex received stronger input related to the sensory features of odors, while posterior regions received stronger input from higher-order associative regions, such as orbitofrontal cortex and basolateral amygdala concerning the meaning of odors (Calu et al., 2007). Our results show that compared to PirF, PirT generally exhibits stronger and more extensive structural connections with the entire brain. In addition to the previous findings, our findings suggest that PirT has exclusive and robust connections to the hippocampus, parahippocampus, and the middle and inferior parts of the temporal lobe, indicating that PirT may play a more significant role in memory processes. It provides novel evidence supporting the suggested distinct functions between PirT and PirF (see Gottfried, 2010 for a review).

TUB is a structure located within the striatum and is part of the mesolimbic dopamine pathway. Non-human animal studies have shown that TUB receives direct input from the olfactory bulb (Scolt et al., 1980), and has efferent projections to several brain areas, especially reward-associated brain regions including pallidum, hypothalamus, amygdala and midbrain (Wesson, 2020; Yamaguchi, 2024; Zhang et al., 2017). We found that TUB is primarily connected to the subcortical nuclei, including the hippocampus, caudate, pallidum, thalamus, amygdala, and putamen, consistent with its proposed role in olfactory reward processing in an optogenetic study (Midroit et al., 2021). Unlike other sensory systems, odor processing occurs directly in the POC without first relaying through the thalamus (Gottfried, 2010; Shepherd, 2005). However, olfactory cognitive tasks sometimes elicit activity in the thalamus (Eek et al., 2023; Plailly et al., 2008). Our observed strong connection between the TUB and the thalamus suggests that the thalamus may also play a role in the olfactory system, potentially facilitating the transfer of information between the POC and cortical regions involved in higher-order processing (Courtiol & Wilson, 2014).

AON is located in the most rostral part of the POC, and showed strong connections to the orbital frontal cortex, including the rectus gyrus. The current finding of a strong structural connectivity between the AON and the orbitofrontal cortex aligns with previous anatomical tracing studies in rodents (Illig, 2005) and human functional connectivity studies (Zhou et al., 2019). The orbitofrontal cortex is involved in odor-based spatial memory and navigation (Dahmani et al., 2018), odor identification and other knowledge-based multisensory integration processes (Olofsson et al., 2014; Pierzchajlo et al., 2024) and might play a critical role in olfactory consciousness (Li et al., 2010). Our findings reveal a strong connection between the POC and the frontal orbital cortex, supporting the evolutionary perspective that animals heavily rely on olfaction to navigate, search for food, and avoid predators (Jacobs, 2012; Stevenson, 2009), but also that olfaction relies on multisensory integration for source-based identification of odor sources in variable and “noisy” chemosensory environments (Pierzchajlo & Olofsson, 2023; Rokni & Ben-Shaul, 2024; Wilson & Stevenson, 2003).

### 4.2 Older adults show weaker structural connectivity of the olfactory network

Our results are based on a participant pool that varied widely in age, thus making our data suitable for investigating age effects on the olfactory network. Previous studies have found that there is an age-related volume loss of the olfactory bulb and other brain regions within the olfactory network, including amygdala, anterior olfactory nucleus, and frontal poles in older adults (Kurth et al., 2019; Shen et al., 2013; Segura et al., 2013). Our study extends these findings by showing weaker structural connectivity strength among these regions (i.e., POC-hippocampus, caudate-TUB, caudate-PirF, amygdala-TUB, and insula-PirT connections) in older compared to younger adults.

In addition to these local alterations, we also found weak positive age effects of the POC subregions with distant brain areas, such as the superior frontal, superior occipital, cingulate, and superior and inferior parietal cortices. In addition to the typical observation of lower white-matter integrity (e.g., Sexton et al., 2014), higher white-matter integrity in older adults has also been repeatedly reported, but not fully discussed in previous cross-sectional and longitudinal DTI studies (Bennett et al., 2010; Burzynska et al., 2010; Sexton et al., 2014). The increased long-distance connections in the olfactory network could align with previously reported whole-brain trends of reduced segregation with age (Chan et al., 2014; Coelho et al., 2021; Li et al., 2023). However, there is currently no post-mortem data supporting such results reported in MR diffusion studies. The results may also reflect artifacts during scanning, imaging processing, or tractography, and should therefore be interpreted with caution.

### 4.3 Structural connection of the olfactory network supports both memory and olfactory functions

Although most human olfactory behaviors are intrinsically driven by multisensory interactions, the underlying structural brain connectivity networks are largely unknown. Focusing on individual brain regions, previous lesion and volumetric studies found that the hippocampus, amygdala, and orbital frontal lobe were involved in successful odor identification (Frasnelli et al., 2010; Jones-Gotman & Zatorre, 1988; Menelaou et al., 2022; Segura et al., 2013). In this study, we applied an optimized white-matter model and by taking advantage of KRR, a multivariate approach, we were able to feed the whole olfactory network into the model to uncover the more complex structural foundation of human olfaction. Our results extend previous findings by showing that the connectivity strength of AON-hippocampus, TUB-hippocampus, TUB-caudate, TUB-putamen, TUB-temporal pole, and TUB-amygdala are critical for successful odor identification. Moreover, the feature-importance maps of age are highly correlated with that of odor identification. This finding indicates that age-related alteration in the olfactory network may explain age-related impairments in odor identification.

Results from the odor threshold test were indicative of individual differences in olfactory sensitivity. Here, we identified specific structural connections crucial for predicting odor thresholds, such as PirT-insula, PirF-amygdala, and PirF-thalamus. Notably, these connections largely differed from those predicting episodic memory and odor identification. Although speculatively, this finding may indicate that the connection with insula, amygdala, and thalamus, which has been linked to self-awareness and emotion, rather than POC-hippocampus connections associated with episodic memory and odor identification, play a role in determining odor thresholds.

Interestingly, we found that the connectome that predicts olfaction of both odor identification and threshold can also predict episodic memory performance. Odors can evoke more vivid and emotional memories compared to other sensory stimuli (Chu & Downes, 2002; Herz & Schooler, 2002; Hinton & Henley, 2013; Larsson & Willander, 2009). Moreover, episodic memory and olfactory function often exhibit similar trajectories in aging (Dintica et al., 2019; Olofsson et al., 2016). This is commonly explained by direct projection between the POC, the hippocampus, and the amygdala that is mainly involved in episodic memory and emotion. However, structural evidence to support this hypothesis has been scarce in humans. The current study provides robust evidence that odor identification and episodic memory are supported by a shared network of connections, including AON-hippocampus, TUB-caudate, and TUB-amygdala connections, thus validating and extending the previous work.

### 4.4 Limitations

The current study has several novel aspects, such that it parcellated POC into subregions, tracked the white matter fibers, and reconstructed the connectome between the POC subregions and the entire brain. However, this approach also requires precise alignment between diffusion imaging and T1 images. Our data was collected with 1000 b-values and 32 directions, which may affect the data quality. Also, diffusion images often suffer from geometric distortion due to B0 inhomogeneity in EPI data, especially in the ventral prefrontal and medial temporal lobe at long echo times such as the one used in the current study (84.4 ms). The current dataset also lacks inversed-phased encoding images, which are typically used to correct the geometric distortion in diffusion images. To overcome these challenges, we employed Synb0, a novel deep-learning algorithm designed to correct the distortion using B0 imaging (Schilling et al., 2019). Additionally, we utilized BBR (Greve & Fischl, 2009), which is robust to a range of pathologies and artefacts in the EPI through additionally aligning the white-matter boundaries between T1 and diffusion images. The final aligned images were also checked individually to ensure the accuracy of the alignment process. To further reduce noise introduced by processing and to enhance statistical power, the resulting connectivity matrix was averaged across the left and right hemispheres, as we had no specific hypothesis regarding lateralization of olfactory networks. However, this approach does not allow for the analysis of connectivity between the left and right POC. Future studies may address this limitation and validate the findings with higher-quality data.

This study is based on a large sample with a wide age range, making it particularly well-suited for analyzing age effects. However, we controlled for age as a confounder in investigating the relationship between the olfactory network and olfactory behavior. The residual variable of olfactory behaviors, obtained after regressing out age, exhibits a small variance, resulting in a relatively low predictive model performance. Despite this limitation, model performance is still better than chance level, allowing for further exploration of predictive feature importance. Future studies with larger samples in the same age range are needed to replicate our results.

In conclusion, by reconstructing white matter connectivity between subregions of the POC and the entire brain and employing multivariate data analytical methods with cross-validation, our study illustrates age-related changes in the organization of the olfactory network. This reorganization may account for the diminished ability of older adults in odor identification. Furthermore, our findings indicate that the olfactory network is capable of predicting both odor threshold and odor identification, as well as episodic memory performance. This study offers new insights into the neural mechanisms underlying age-related olfactory decline and has significant implications for understanding the shared structural foundations of episodic memory and olfaction.

## Supplementary Material

Supplementary Material

## Data Availability

The data that support the findings of this study are available on request from the corresponding author. Due to data protection concerns, publicly sharing the entire data set underlying this study is not possible at the moment.
