# Supplementary material for: Structural connectivity of the human olfactory network and its relation to aging and olfactory function
Source: Imaging Neurosci (Camb). 2026 Mar 27;4:IMAG.a.1181. doi: 10.1162/IMAG.a.1181 (PMC13034614; doi:10.1162/IMAG.a.1181)
Supplement: Supplementary Material [file IMAG.a.1181_supp.pdf]

Supplementary

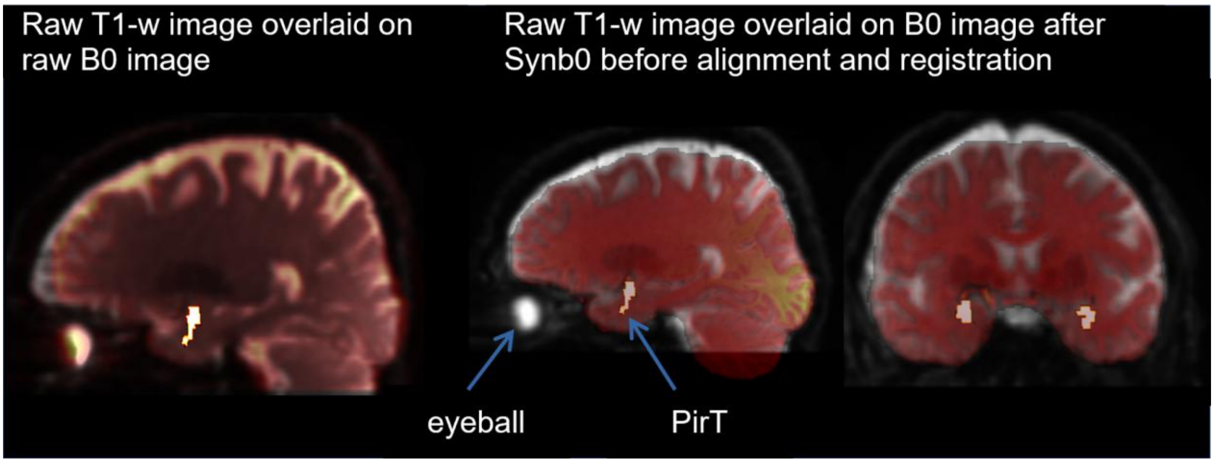

Supplementary Fig. 1. The raw T1-weighted image overlaid on raw (left) and Synb0 processed (middle and right) images. The image shown is from the same young adult participant as in Fig. 1. Geometric distortion especially affects the frontal pole and alters the shape of the eyeballs. For reference, the location of the Piriform Cortex (PirT) is highlighted in the images. Synb0 successfully corrects for this distortion, demonstrating good alignment with the T1-weighted image.

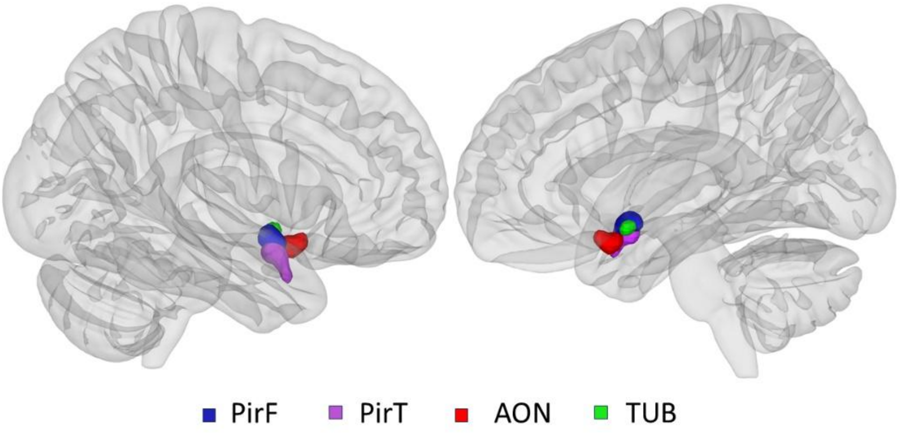

Supplementary Fig. 2. Illustration of POC ROIs used in the current analysis. The figure was generated using the code in (Zhou et al., 2019) https://github.com/elifesciences-publications/primaryolfactorycortexparcellation/

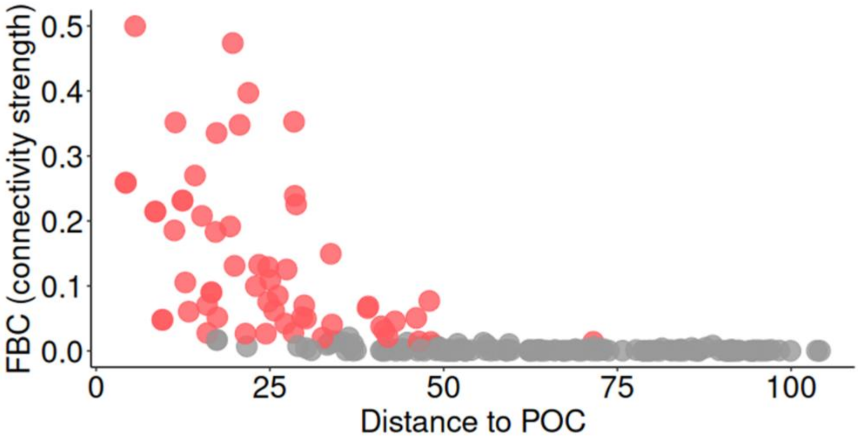

Supplementary Fig. 3. Linear relation between FBC and Euclidean distance. Dots represent connections between POC subregions to other brain regions. The strongly-connected olfactory network (red) mainly includes short-range connections that are close to the POC (See methods for more details regarding how the network is defined).

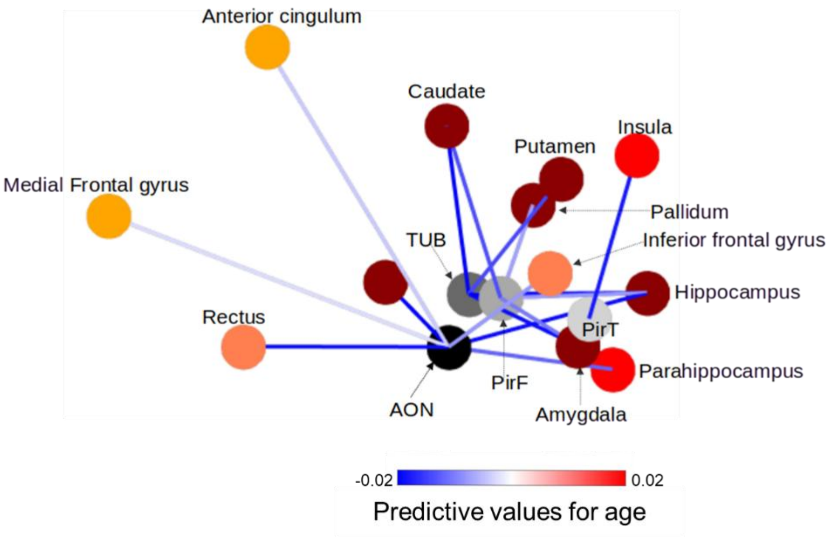

Supplementary Fig. 4 Anatomical labels of the connected ROIs exhibiting negative age effects ($P^{perm} < 0.05$). Edge color represents the predictive-feature values of the connections. Node colors represent the number of connections (0–4; yellow to dark red) to POC subregions within the strongly connected network (See methods for more details regarding how the network is defined).

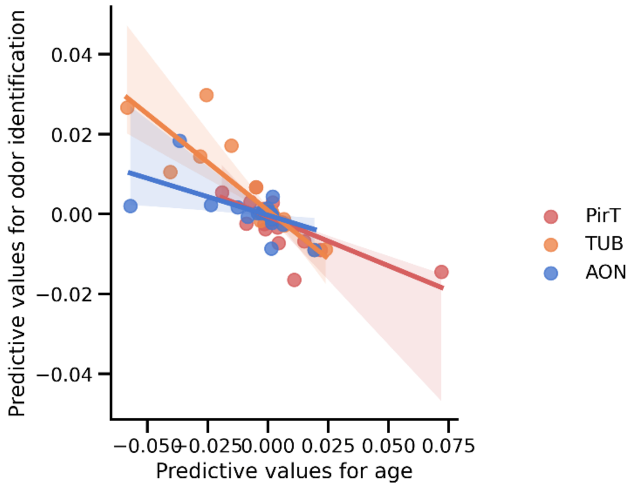

Supplementary Fig. 5 Scatter plot showing the linear relationship between predictive-feature values for age and for odor threshold after controlling for episodic memory. POC subregions showing non-significant predictive correlations ($P^{perm} > 0.05$) of predictive values were omitted for clarity.
